# Comparison of performance characteristics between high‐performance liquid chromatography and latex agglutination turbidimetric immunoassay for therapeutic drug monitoring of zonisamide

**DOI:** 10.1002/jcla.22940

**Published:** 2019-06-20

**Authors:** Daiki Eto, Ryota Tanaka, Yosuke Suzuki, Yuhki Sato, Hiroki Itoh

**Affiliations:** ^1^ Department of Clinical Pharmacy Oita University Hospital Yufu‐shi, Oita Japan

**Keywords:** antiepileptic, high‐performance liquid chromatography, latex‐enhanced turbidimetric immunoassay, therapeutic drug monitoring, zonisamide

## Abstract

**Background:**

Recently, the Nanopia^®^ TDM Zonisamide reagent using the latex particle‐enhanced turbidimetric immunoassay (LTIA) method was developed. The aim of this study was to compare the differences in serum zonisamide (ZNS) concentrations quantified by the high‐performance liquid chromatography (HPLC) method and the LTIA method using a TBA‐25FR analyzer.

**Methods:**

A total of 78 samples from 33 patients were quantified by both HPLC and LTIA methods. Deproteinization was used as pretreatment for the HPLC method. The ZNS concentrations quantified by two methods were compared.

**Results:**

The HPLC method had intra‐ and inter‐day precision lower than 1.86% and 9.00%, and accuracy better than 2.44% and 6.33%, respectively. The LTIA method showed intra‐ and inter‐day precision lower than 2.50% and 5.20%, and accuracy better than 15.80% and 10.60%, respectively. The lower limits of quantification for the HPLC and LTIA methods were 1.0 and 5.0 µg/mL, respectively. The ZNS concentration quantified by the HPLC method correlated strongly with that by the LTIA method (*r* = 0.953, *P* < 0.001). A Bland‐Altman plot suggested no systematic error between ZNS concentrations quantified by HPLC and LTIA methods.

**Conclusion:**

This study confirmed no differences between the concentrations quantified by the HPLC and LTIA methods at both high and low concentrations, demonstrating the confidence of measurement by the LTIA method.

## 1 INTRODUCTION

Zonisamide (ZNS) is one of the second‐generation antiepileptic drugs with potent and broad‐spectrum activities against simple and complex partial seizures, secondary generalized tonic‐clonic convulsions, atypical absence seizures, general seizures, atypical petit mal, and mixed seizures.^1^, ^2^ This drug can be used to treat the above seizures in adults and children. The mechanism of antiepileptic action is thought to involve blocking of voltage‐sensitive sodium channels and voltage‐sensitive T‐type calcium channels, inducing stabilization of neuronal membranes and suppression of neuronal hypersynchronization.^2^, ^3^

Orally administered ZNS is rapidly and completely absorbed, reaching peak plasma concentrations after 2‐5 hours in healthy adult volunteers.^4^ Steady‐state serum levels are reached after 10‐12 days. Although ZNS has been reported to exhibit nonlinearity between dosage and serum concentration,^5^ linear pharmacokinetics have been demonstrated in the therapeutic concentration range in steady state.^6^ ZNS is metabolized via phase I and phase II biotransformation pathways, and the metabolites have no activity.^7^ Since ZNS is metabolized by cytochrome P450 (CYP)3A4, other drugs that induce or inhibit this enzyme would induce or inhibit ZNS metabolism. Actually, CYP3A4 inducers reduce the plasma half‐life of ZNS from approximately 60‐30 hours, while inhibitors such as cimetidine, erythromycin, and ketoconazole may prolong the half‐life.^7^ Therefore, therapeutic drug monitoring (TDM) of ZNS is recommended not only when response is inadequate or adverse effects occur, but also when drug‐drug interaction is a concern. A target plasma concentration range of 10‐40 µg/mL has been recommended for seizure management.^8^ When the blood concentration exceeds 40 µg/mL, adverse effects such as drowsiness and impaired attention tend to occur.^9^

Many quantitative methods using high‐performance liquid chromatography (HPLC) have been established to measure plasma ZNS concentration either alone or simultaneously with other drugs.^10^, ^11^, ^12^, ^13^, ^14^, ^15^, ^16^ Until now, we have also used the HPLC method for determination of ZNS levels. Recently, the Nanopia^®^ TDM Zonisamide reagent using the latex particle‐enhanced turbidimetric immunoassay (LTIA) method was developed. The assay can be performed using a fully automated TBA‐25FR analyzer and is characterized by high speed and versatility. However, there is no report on the correlation between ZNS concentrations measured by the HPLC and LTIA methods.

The aim of this study was to compare serum ZNS concentrations measured by the HPLC method that we developed previously and by the LTIA method using a TBA‐25FR analyzer.

## 2 MATERIALS AND METHODS

### 2.1 Materials

Zonisamide and zonisamide N,N‐dimethylformimidamide were purchased from Wako Pure Chemical Industries. COSMOSIL 5C18‐MS‐II packed column (4.6 × 150 mm) was purchased from nacalai tesque. Nanopia^®^ TDM Zonisamide, Zonisamide Calibrator, and Zonisamide Control were purchased from Sekisui Medical. All other chemicals were of the highest grade commercially available. All solutions were prepared using deionized and distilled water.

### 2.2 Subjects

Patients who were given ZNS to treat epilepsy and underwent routine TDM at Oita University Hospital between November 2015 and January 2017 were enrolled in this study. Blood samples were collected in serum separator tubes and centrifuged at 1900 *g* at 4°C for 5 minutes. After centrifugation, serum samples were separated. This study was approved by the ethics committee of Oita University (approval number: 664). Informed consent was not necessary because blood samples were collected for routine monitoring and assessment of therapeutic agents as a part of regular patient care.

### 2.3 HPLC method

Stock solutions of ZNS and ZNS N,N‐dimethylformimidamide at concentrations of 200 and 10 µg/mL, respectively, were prepared by weighing in separate measuring flasks and dissolving in 100% methanol. Stock solutions were stored at −40°C. To prepare calibrating solutions at concentrations of 1, 2, 5, 10, 20, 50, and 100 µg/mL, the stock solution of ZNS was diluted in 100% methanol, then evaporated to dryness by N_2_ gas at 40°C, and redissolved in 250 µL of blank serum. Lower limit of quantification (LLOQ), and low, medium, and high quality control (QC) solutions at concentrations of 0.5, 1.5, 15, and 75 µg/mL, respectively, were prepared by the same procedure. One hundred microliter of solution prepared above was dispensed into a 2‐mL polypropylene tube and deproteinized by adding 200 µL of 100% methanol containing ZNS N,N‐dimethylformimidamide as internal standard. After centrifugation at 15 000 *g* at 4°C for 5 minutes, 200 µL of the supernatant was collected. Then, 15 µL of the supernatant was injected into HPLC.

Samples were analyzed using a Waters e2695 Separation Module equipped with a Waters 2489 UV/visible detector. The analytes were detected at 238 nm. Chromatographic separation of the analytes was performed using a COSMOSIL 5C18‐MS‐II packed column (4.6 × 150 mm). The mobile phase for chromatographic separation consisted of 1% aqueous acetic acid, isopropyl alcohol, and acetonitrile (70:11:10). The column temperature was maintained at 40°C throughout all experiments, whereas the sample temperature was maintained at 20°C. The total analysis time was 10 minutes per injection, and the flow rate was 0.5 mL/min. Peak areas of the respective analytes were divided by those of internal standard, and calibration curves for ZNS measurement were constructed.

### 2.4 LTIA method

A TBA™‐25FR analyzer was used for ZNS determination by the LTIA method. According to the manufacturer's information for Nanopia^®^ TDM, the concentrations of calibration solutions were 5, 10, 20, 40, and 80 μg/mL. The concentrations of LLOQ, and low, medium, and high QC solutions were 3, 8, 25, and 50 μg/mL, respectively. The total analysis time was approximately 15 minutes per measurement (simultaneous measurement of multiple sample is possible).

### 2.5 Validation of the two measurement methods

Using both methods, QC samples at different concentrations (LLOQ and low, medium, and high QCs) were measured in sextuplicate and the results were analyzed for intra‐day precision and accuracy. Measurements of the same QC samples in triplicate were performed there times on different days, and the results were analyzed for inter‐day precision and accuracy. Precision and accuracy were calculated and expressed as coefficient of variation (CV%) and relative error (RE%), respectively, for each intra‐day and inter‐day assay.

### 2.6 Correlation between measurements by HPLC and LTIA methods

Serum ZNS concentrations of all patients were quantified using both HPLC and LTIA methods, and the measurements were compared. Correlation between serum ZNS concentrations measured by the two methods was analyzed using Spearman's rank correlation coefficient. Statistical analyses were carried out using IBM SPSS Statistics 23 (IBM Corporation).

## 3 RESULTS

### 3.1 Patients characteristic

Thirty‐three patients comprising 22 males and 11 females were enrolled in this study. The median age at the time of enrollment was 32 years (range 0‐82 years). A total of 78 samples were analyzed. The median daily dose was 200 mg (range 80‐600 mg).

### 3.2 Validation result of the HPLC method

**Figure 1:**
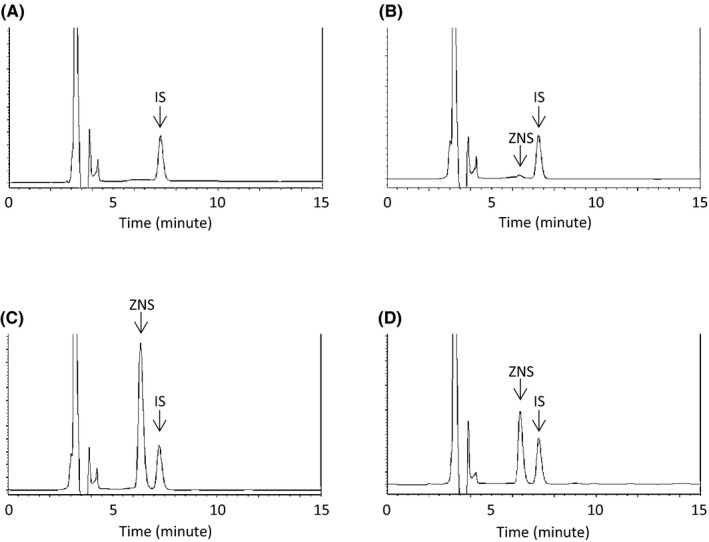
Chromatograms of (A) blank serum; (B) serum spiked with LLOQ at concentration of 1.0 µg/mL; (C) serum spiked with high QC at concentration of 75 µg/mL; (D) serum sample of a patient. LLOQ, lower limit of quantitation; QC, quality control

Figure 1 shows the chromatographic characteristics of ZNS and ZNS N,N‐dimethylformimidamide for blank serum, LLOQ, high QC, and serum sample of a patient who received ZNS. Under the optimized chromatographic conditions, the retention times of ZNS and ZNS N,N‐dimethylformimidamide were 6.4 and 7.3 minutes, respectively. LLOQ fulfilled the FDA guidance criteria (precision: below 20%, accuracy: between −20% and 20%) for both intra‐ and inter‐day precision (1.17% and 10.27%, respectively) and accuracy (3.73% and −5.78%, respectively) (Table 1). Intra‐day precision of the three QCs was <1.86%, and accuracy was better than 2.44%, while inter‐day precision was <9.00% and accuracy was better than 6.33%. All QCs also fulfilled he FDA guidance criteria for both intra‐ and inter‐day precision (below 15%) and accuracy (between −15% and 15%).

**Table 1:** Validation results for measuring zonisamide concentrations in human serum by the HPLC method

	Zonisamide concentrations measured by HPLC (µg/mL)			
	LLOQ	QC A	QC B	QC C
	1	3	30	75
Intra‐day				
Mean (µg/mL)	1.04	3.02	30.18	73.17
Accuracy (%)	3.73	0.59	0.59	−2.44
Precision (%CV)	1.17	1.33	1.86	1.57
Inter‐day				
Mean (µg/mL)	0.94	2.93	28.81	70.25
Accuracy (%)	−5.78	−2.45	−3.98	−6.33
Precision (%CV)	10.27	4.95	6.39	9.00

Abbreviations: CV, coefficient of variation; HPLC, high‐performance liquid chromatography; LLOQ, lower limit of quantitation; QC, quality control.

### 3.3 Validation result of the LTIA method

According to manufacturer's information for Nanopia^®^ TDM, the concentration of LLOQ was 3 μg/mL. However, measurements at this concentration did not fulfill the FDA guidance criteria of accuracy (between −25% and 25%) in this study (data not shown). Thus, we changed the LLOQ of the LTIA method in our study from 3 to 5 μg/mL, and the LLOQ of 5 μg/mL fulfilled the FDA guidance criteria for both intra‐ and inter‐day precision (3.56% and 2.48%, respectively) and accuracy (4.67% and 6.67%, respectively) (Table 2). Intra‐day precision of three QCs was <2.50% and accuracy was better than 15.80%, while inter‐day precision was <5.20% and accuracy was better than 10.60%.

**Table 2:** Validation results for measuring zonisamide concentrations in human serum by the LTIA method

	Zonisamide concentrations measured by LTIA (µg/mL)			
	LLOQ	QC A	QC B	QC C
	5	8	25	50
Intra‐day				
Mean (µg/mL)	5.23	6.70	22.60	45.20
Accuracy (%)	4.67	−15.80	−9.50	−9.50
Precision (%CV)	3.56	1.80	2.50	2.40
Inter‐day				
Mean (µg/mL)	5.33	7.20	23.60	46.20
Accuracy (%)	6.67	−10.60	−5.70	−7.60
Precision (%CV)	2.48	5.20	3.70	2.20

Abbreviations: CV, coefficient of variation; LLOQ, lower limit of quantitation; LTIA, latex‐enhanced turbidimetric immunoassay; QC, quality control.

### 3.4 Correlation of measurements between the HPLC and LTIA method

**Figure 2:**
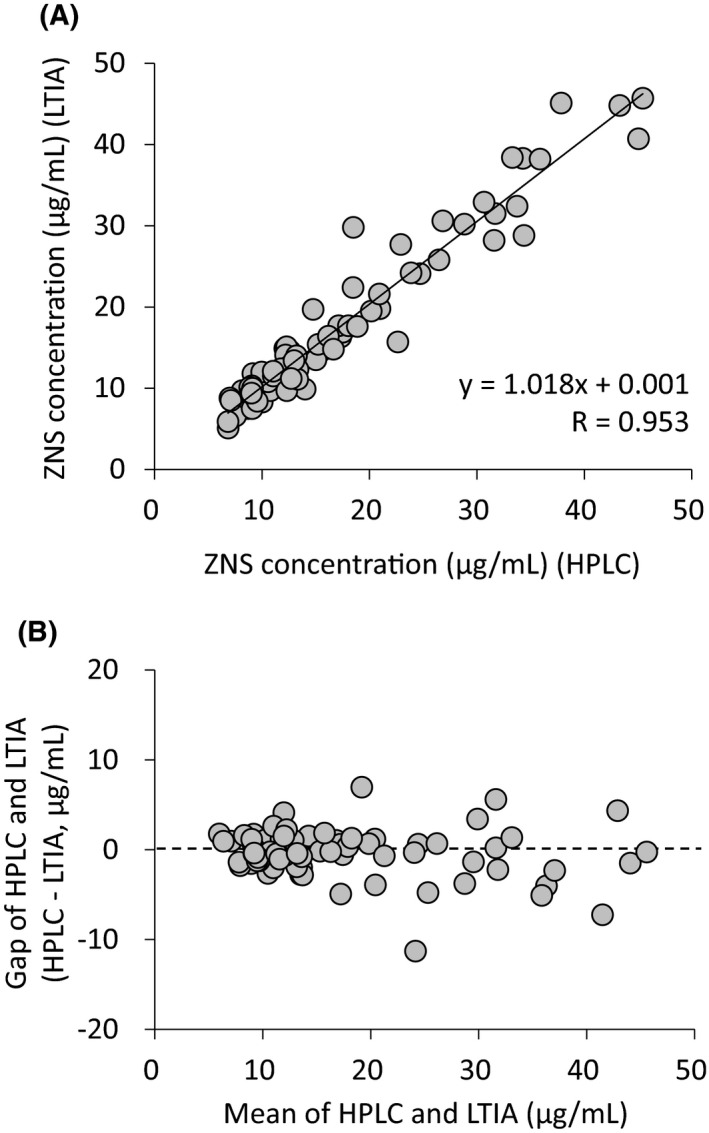
Relationship between serum zonisamide concentrations measured by HPLC and LTIA methods (A), and a Bland‐Altman plot for serum zonisamide concentrations measured by HPLC and LTIA methods (B). HPLC, high‐performance liquid chromatography; LTIA, latex‐enhanced turbidimetric immunoassay

Serum ZNS concentrations of 78 samples from the patients were quantified using the HPLC and LTIA methods. Four serum samples had serum concentrations lower than LLOQ when measured by the LTIA method, while the serum concentrations of all samples ranged within the standard curve when measured by the HPLC method. Seventy‐four of 78 samples that had concentrations higher than LLOQ by the LTIA method were used in the analysis. Serum ZNS concentration measured by the HPLC method correlated strongly with that by the LTIA method (*r* = 0.953, *P* < 0.001) (Figure 2A). To confirm the dispersion between the two methods, we constructed a Bland‐Altman plot using serum ZNS concentrations measured by HPLC and LTIA. The result suggested no systematic error between ZNS concentrations measured by the two methods (Figure 2B).

## 4 DISCUSSION

In the HPLC method for ZNS quantification, deproteinization was used in the pretreatment procedure, and the LLOQ and calibration curve range were set at 1 and 1‐80 μg/mL, respectively. There are some reports on the quantification of ZNS in serum or plasma using HPLC method.^10^, ^11^, ^12^, ^13^, ^14^, ^15^, ^16^ Greiner‐Sosanko et al^10^ developed a HPLC method for simultaneous determination of ZNS, lamotrigine, and carbamazepine, using pretreatment by liquid‐liquid extraction under alkaline conditions into an organic solvent. Additionally, some HPLC methods previously developed to quantify ZNS concentration pretreated samples by solid‐phase extraction.^11^, ^12^, ^15^ Pretreatment by liquid‐liquid extraction or solid‐phase extraction allows more complete removal of serum or plasma matrix than deproteinization. Liquid‐liquid extraction or solid‐phase extraction is superior to deproteinization in terms of sensitivity and specificity, making it possible to set lower LLOQ and quantify many drugs simultaneously. However, liquid‐liquid extraction requires an evaporation to dryness step, while solid‐phase extraction requires many steps such as column conditioning, equilibration, application, washing, and elution. Since the steps are time‐consuming and involve complicated procedures, the use of liquid‐liquid extraction or solid‐phase extraction may be less suitable for routine TDM. By contrast, our deproteinization step only requires addition of methanol containing internal standard to serum followed by centrifugation, which is simple and takes little time for pretreatment.

The simple method of ZNS quantification using deproteinization as the pretreatment procedure was reported by Yoshida et al^13^ and Contin et al^16^ However, the LLOQs of their quantification methods were 2.5 and 2.0 µg/mL, respectively, which are higher than that of our HPLC method. In this study, serum ZNS concentrations of 4 samples could not be measured by LTIA method because they were lower than the LLOQ (5 µg/mL), and the concentrations quantified by our HPLC method were 1.4, 3.5, 2.9, and 2.1 µg/mL. The methods of Yoshida et al^13^ and Contin et al^16^ would not be able to determine the concentrations of 2 samples and 1 sample, respectively, whereas our HPLC method was able to measure all samples. Quantification of ZNS in the low concentration range is generally not considered to be important, because the target range is 10‐40 µg/mL. Nonetheless, determination of lower concentrations may be useful when a low concentration of ZNS has to be maintained during combination therapy with other antiepileptic drugs and when confirming compliance. Our HPLC method has the advantage of being able to measure lower concentrations using a simple pretreatment procedure. The procedure of the LTIA method is simple and easy to perform because no pretreatment is required. Hence, we compared the performance of our HPLC method with that of LTIA method.

Although determination of ZNS using competitive binding enzyme immunoassay was developed previously,^17^ there is no report on the LTIA method. Immunoassays including LTIA utilize antigen‐antibody reactions. Hence, false‐positive reactions may be caused by reactions of structurally similar substances such as metabolites.^18^ On the other hand, a few reports have described false‐negative reactions due to masking of antibody by high concentration of immunoglobulin M.^19^, ^20^ As ZNS is metabolized primarily by CYP3A4, the possibility of false‐positive reactions due to metabolites cannot be ruled out. As shown in Figure 2A, the ZNS concentration measured by the HPLC method correlated strongly with that determined by the LTIA method. The slope and intercept of the regression line were 1.018 and 0.001, respectively, suggesting the possibility that both false‐positive and false‐negative reactions were absent when assayed by the LTIA method. Furthermore, fixed error and proportional error were not observed between ZNS concentrations measured by the two methods, indicating the validity of ZNS concentrations measured by the LTIA method (Figure 2B).

For the LTIA method used in this study, the LLOQ that fulfilled the criteria of FDA guidance was 5 µg/mL. As mentioned above, the target range of ZNS is 10‐40 µg/mL. The LTIA method may not be able to determine the exact concentration of ZNS in patients in whom serum level is maintained below the target range, or may not be used in TDM for the purpose of confirming compliance. Thus, the HPLC method will have to be used to confirm the exact levels when the LTIA method shows concentrations below 5 µg/mL.

In conclusion, the present study confirms no difference in serum ZNS concentrations quantified by the HPLC and LTIA methods at both high and low concentrations, demonstrating the confidence of ZNS measurement using the LTIA method.
